# Cooperation of Complement MASP-1 with Other Proinflammatory Factors to Enhance the Activation of Endothelial Cells

**DOI:** 10.3390/ijms24119181

**Published:** 2023-05-24

**Authors:** Zsuzsanna Németh, Márta L. Debreczeni, Erika Kajdácsi, József Dobó, Péter Gál, László Cervenak

**Affiliations:** 1 Department of Internal Medicine and Haematology, Semmelweis University, 1085 Budapest, Hungary; nemethzsanna@gmail.com (Z.N.);; 2 Research Group for Immunology and Haematology, Semmelweis University—Eötvös Loránd Research Network (Office for Supported Research Groups), 1052 Budapest, Hungary; 3 Institute of Enzymology, Research Centre for Natural Sciences, 1117 Budapest, Hungary

**Keywords:** MASP-1, endothelial cell, cooperation, LPS, IFNγ, histamine, bradykinin, inflammation, synergy, in vitro

## Abstract

Endothelial cells play an important role in sensing danger signals and regulating inflammation. Several factors are capable of inducing a proinflammatory response (e.g., LPS, histamine, IFNγ, and bradykinin), and these factors act simultaneously during the natural course of the inflammatory reaction. We have previously shown that the complement protein mannan-binding lectin-associated serine protease-1 (MASP-1) also induces a proinflammatory activation of the endothelial cells. Our aim was to investigate the possible cooperation between MASP-1 and other proinflammatory mediators when they are present in low doses. We used HUVECs and measured Ca^2+^ mobilization, IL-8, E-selectin, VCAM-1 expression, endothelial permeability, and mRNA levels of specific receptors. LPS pretreatment increased the expression of PAR2, a MASP-1 receptor, and furthermore, MASP-1 and LPS enhanced each other’s effects in regulating IL-8, E-selectin, Ca^2+^ mobilization, and changes in permeability in a variety of ways. Cotreatment of MASP-1 and IFNγ increased the IL-8 expression of HUVECs. MASP-1 induced bradykinin and histamine receptor expression, and consequently, increased Ca^2+^ mobilization was found. Pretreatment with IFNγ enhanced MASP-1-induced Ca^2+^ mobilization. Our findings highlight that well-known proinflammatory mediators and MASP-1, even at low effective doses, can strongly synergize to enhance the inflammatory response of endothelial cells.

## 1. Introduction

The endothelial lining of our circulatory system plays a role in several important physiological processes by regulating the blood pressure, hemostasis, and leukocyte homing and controlling vascular permeability. Endothelial cells (ECs) act as signal integrators; in other words, they have numerous cell surface receptors, adhesion molecules, and a well-developed cytoskeleton system to respond adequately to various molecules present in the blood [1]. As they are in direct contact with blood, the activation of plasma cascade systems, cytokines, chemokines, proteases, and other molecules produced by immune and non-immune cells in the blood directly and simultaneously affect their behavior. These factors belong to different parts of the immune system, and their combined presence and/or activation are characteristic of different diseases and pathological conditions.

Mannan-binding lectin-associated serine protease 1 (MASP-1) is one of the key components of the complement lectin pathway [2]. Mannan-binding lectin (MBL), collectins 10 and 11, and ficolins (H-, L-, and M) circulate in the blood in complexes with associated serine proteases (MASP-1 and -2) and recognize pathogens or altered host cells [3]. The recognition leads to the autoactivation of MASP-1, which activates MASP-2, initiating the lectin pathway. Other than this, MASP-1 can cleave several other molecules, e.g., HMWK, fibrinogen, factor XIII, and TAFI [4]. MASP-1 activates ECs by the cleavage of protease-activated receptors (PARs), namely PARs 1, 2, and 4 [5]. This activation induces the ECs to enter a pro-inflammatory state in several ways: activation of pro-inflammatory genes [6] and signaling pathways [5], expression of inflammatory cytokines [7] and adhesion molecules [8], and increased permeability [9].

Lipopolysaccharide (LPS) is an endotoxin, a molecule from the Gram-negative bacteria outer membrane, which triggers serious inflammatory reactions in the human body via the LBP/CD14/TLR4/MD2 receptor complex [10]. LPS increases endothelial permeability, triggers superoxide release, and enhances adhesion molecule expression and inflammatory cytokine production [11,12,13].

Histamine is the major mediator of acute allergic reactions, released from mast cells and basophil granulocytes [14]. Histamine acts on four receptors (H1R–H4R) with different tissue distributions and effects, but most notably, in the vascular system, it rapidly increases endothelial permeability through H1R [15,16].

Bradykinin is a small oligopeptide liberated from high-molecular-weight kininogen (HMWK) by plasma serine proteases (mainly plasma kallikrein and, to a smaller extent, other enzymes, including MASP-1) [17] and acting on two receptors (B1R, B2R) [18]. Bradykinin is a potent vasodilator, promotes inflammation, and stimulates the sensory nerves, which leads to hyperpermeability [19,20].

IFNγ is a cytokine produced predominantly by T_H_1, T_C_1, and NK cells during viral infections. IFNγ has two receptors (IFNGR1 and 2), and it is an important activator of macrophages and an inducer of Class II major histocompatibility complex (MHC) molecule expression. It activates the inducible nitric oxide synthase (iNOS), promotes adhesion and leukocyte migration, and increases endothelial permeability [21].

The complement system is activated, among others, during bacterial [22], viral [23], or fungal [24] infections; it plays an important role in angioedema-causing disorders, such as hereditary angioedema (HAE) [25], and can also participate in allergic reactions [26].

In most in vitro models, the response elicited by a single selected molecule is usually studied. However, the effects of this molecule can be substantially modified if we apply it with a combination of several other relevant substances, and the resulting cellular response can be significant even in low individual concentrations [27]. To model the cooperation of MASP-1 with molecules that have fundamental roles in microbial-, allergic-, and other complement systems involving diseases, we chose four important endothelial cell activators (LPS, IFNγ, bradykinin, and histamine) with different effects. ECs express the receptors for the above-mentioned activators, creating the possibility that we can assess the effects of the potential interaction between these molecules and MASP-1. The cooperation can manifest in several ways, such as the altered expression of cytokines and adhesion molecules, increased or decreased receptor expression, and distinct intracellular signaling or altered permeability.

In a major injury, the activation of the complement pathway and the entry of bacteria or viruses occur almost simultaneously (cotreatment). In a milder infection, when bacteria break through a tissue layer, the activation of the complement pathway in the blood can be a secondary reaction (pretreatment with a coactivator). However, in chronic or prolonged inflammation, the lectin pathway may already be activated, and a secondary infection may result in newly synthesized or incoming activators (pretreatment with MASP-1). The investigation of this cooperation may contribute to a better understanding of the behavior of endothelial cells during inflammatory conditions.

In this study, we present that MASP-1 interacts synergistically with other EC activator molecules to induce an increased proinflammatory response in endothelial cells.

## 2. Results

### 2.1. Experimental Setup

**Figure 1:**
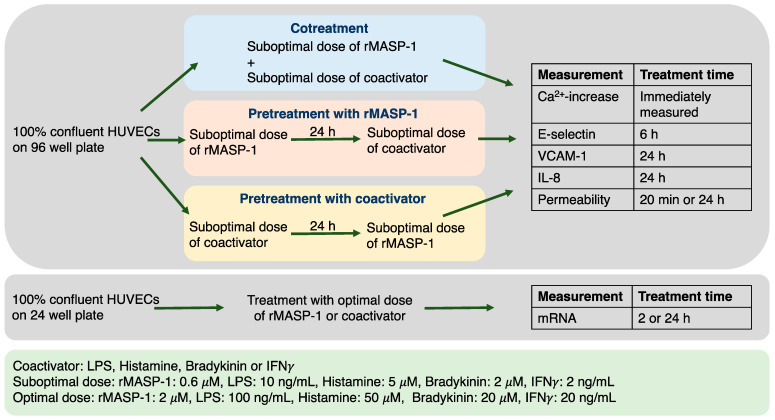
A schematic overview of the experimental setup. The blue, pink, and yellow colors of the boxes representing the experimental setups are used for all subsequent figures for better understanding.

Different proinflammatory activators acting on endothelial cells (among others) can be present simultaneously or sequentially in the circulation during a bacterial (LPS and MASP-1) or viral (IFNγ and MASP-1) infection, in certain diseases such as HAE (bradykinin and MASP-1), or during an allergic reaction accompanied by a bacterial or fungal infection (histamine and MASP-1). Three types of possible interactions were investigated to explore possible cooperation with the complement lectin pathway and other coactivators. Cells were either cotreated with recombinant MASP-1 (rMASP-1) and one of the coactivators, pretreated with rMASP-1 and then treated with one of the coactivators, or treated with rMASP-1 after pretreatment with one of the coactivators. These experimental setups modeled different pathophysiological conditions. The pretreatments always lasted for 24 h, while treatment time varied depending on the parameter measured (Figure 1). Pre-determined [7] suboptimal doses of treatments were used to ensure the possibility of an accurate measurement of the cellular response, even when combined treatments were applied.

We measured cellular responses that were previously shown to be affected by MASP-1, either at the mRNA or protein level in endothelial cells. Intracellular Ca^2+^ mobilization led to general signaling from various activated receptors, including G-protein-coupled protease-activated receptors (PARs), released IL-8 acted as a neutrophil chemoattractant, and adhesion molecules, such as E-selectin and VCAM-1, promoted the rolling and transmigration of leukocytes on endothelial cells, whereas increased vascular permeability ensured an effective extravasation of soluble immunological and acute phase mediators.

Due to the large amount of data generated, in the following section, only those results are presented in detail, where significant cooperation could be found between MASP-1 and the various treatments. Nevertheless, we summarized the data obtained from every experimental setup in Table 1.

### 2.2. rMASP-1 Cooperates with LPS, IFNγ, Bradykinin, and Histamine to Induce Intracellular Ca^2+^ Mobilization

**Figure 2:**
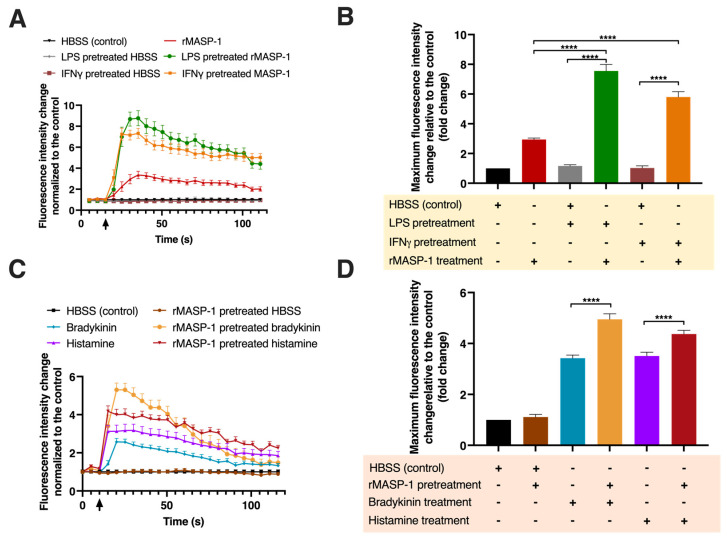
MASP-1 interacted with LPS, IFNγ, histamine, and bradykinin to induce intracellular Ca^2+^ mobilization in HUVECs. Confluent layers of HUVECs were cultured in 96-well plates, and the cells were pretreated with LPS (10 ng/mL), IFNγ (2 ng/mL), bradykinin (2 μM), rMASP-1 (0.6 μM), or histamine (5 μM) for 24 h or with medium alone (control). After the removal of pretreatment, the cells were loaded with 2 μM of Fluo-4-AM. Sequential images were taken by fluorescence microscopy every 5 s. Two images were taken initially to determine baseline fluorescence; then, the treatment was applied, and the response was measured for 2 min. Left-side panels show data from a single, representative experiment, where fluorescence intensity values were background corrected and normalized to the control (**A**,**C**). Diagrams in the right-side panels show the means of maximum fluorescence intensity values from three independent experiments, normalized to the control. (**B**) LPS or IFNγ pretreatment was followed by rMASP-1 treatment. (**D**) rMASP-1 pretreatment was followed by bradykinin or histamine treatment. Compared to the control: HBSS vs. rMASP-1: **, HBSS vs. LPS-pretreated HBSS: ns., HBSS vs. LPS-pretreated rMASP-1: **, HBSS vs. IFNγ-pretreated HBSS: ns., HBSS vs. IFNγ-pretreated rMASP-1: **, HBSS vs. rMASP-1-pretreated HBSS: ns., HBSS vs. bradykinin: ****, HBSS vs. rMASP-1-pretreated bradykinin: ****, HBSS vs. histamine: ***, HBSS vs. rMASP-1-pretreated histamine: ****; +: indicates that the pretreatment or treatment was added; -: indicates that pretreatment or treatment was not added; ↑: indicates the addition of the treatment; ns: non-significant; ** *p* < 0.01; *** *p* < 0.001; **** *p* < 0.0001.

Ca^2+^ mobilization is an important step in the signal transduction of several receptors, such as GPCR-coupled histamine, bradykinin receptors, and PARs. Although LPS or IFNγ themselves did not trigger Ca^2+^ mobilization in HUVECs, pretreatment with either of these factors significantly increased the Ca^2+^ mobilization response to rMASP-1 treatment (Figure 2A). Interestingly, cotreatment with MASP-1 with either BK or HA did not result in an increased Ca^2+^ mobilization however, both BK and HA elicited greater Ca^2+^ mobilization in the HUVECs after rMASP-1 pretreatment (Figure 2B).

### 2.3. rMASP-1 Cooperates with LPS in the Induction of E-selectin Expression

**Figure 3:**
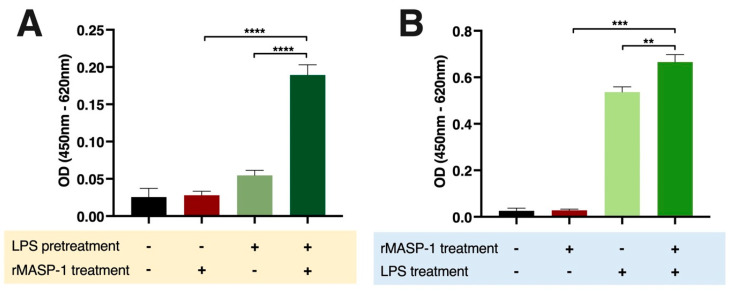
rMASP-1 cooperated with LPS in the induction of E-selectin on endothelial cells. Confluent layers of HUVECs were cultured in 96-well plates. (**A**): The cells were pretreated with LPS (10 ng/mL) for 24 h, and after the removal of the pretreatment, rMASP-1 (0.6 μM) was added to the cells for 6 h. (**B**): In the case of cotreatment, LPS (10 ng/mL) and rMASP-1 (0.6 μM) were added together to the cells for 6 h. Then, the cells were fixed, and the expression level of E-selectin was determined by cell-based ELISA. Compared to the control: control vs. rMASP-1: ns., control vs. LPS-pretreated control: ns., control vs. rMASP-1 treatment after LPS pretreatment: ****, control vs. LPS: ****, control vs. LPS + rMASP-1: ****; +: indicates that the pretreatment or treatment was added; -: indicates that pretreatment or treatment was not added; ns: non-significant; ** *p* < 0.01; *** *p* < 0.001; **** *p* < 0.0001.

We measured the expression of two important adhesion molecules, E-selectin and VCAM-1, indicating proinflammatory changes in the adhesion capacity of endothelial cells to leukocytes. LPS and rMASP-1 interacted in the induction of E-selectin in two different ways. Both the cotreatment and the LPS pretreatment were followed by rMASP-1 treatment and significantly increased the E-selectin levels in HUVECs (Figure 3A,B). We could not find any significant cooperation in the induction of VCAM-1 between rMASP-1 and the other activators.

### 2.4. rMASP-1 Cooperates with LPS, IFNγ, and Histamine in the Induction of IL-8 Production

**Figure 4:**
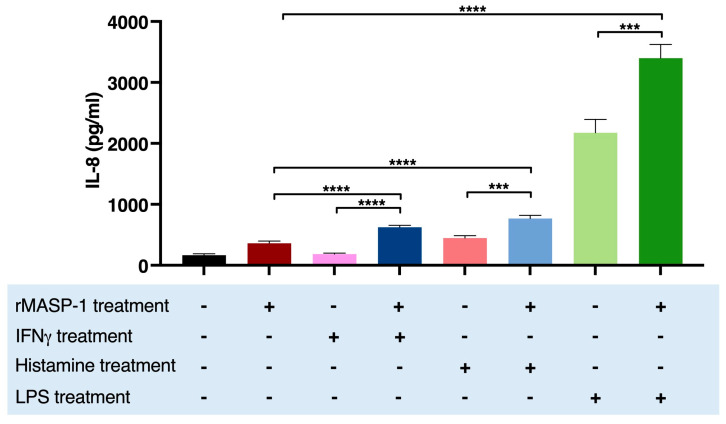
rMASP-1 interacted with IFNγ, histamine, and LPS in the induction of the IL-8 production of HUVECs. Confluent layers of HUVECs were cultured in 96-well plates, and the cells were cotreated with rMASP-1 and LPS, histamine, or IFNγ for 24 h. The supernatants were collected and diluted in 1:20, and then the IL-8 concentration was measured by sandwich ELISA. The results of the ELISAs were calculated from the standard curve and plotted as mean concentration values of three independent experiments. Compared to the control: control vs. rMASP-1: ****, control vs. IFNγ: ns., control vs. histamine: ****, control vs. LPS: ****; +: indicates that the pretreatment or treatment was added; -: indicates that pretreatment or treatment was not added; ns: non-significant; *** *p* < 0.001; **** *p* < 0.0001.

IL-8 is a chemokine secreted by endothelial cells with a low constitutive rate and can be induced by various stimuli. Treatment with LPS [28], histamine, or rMASP-1 [7] alone increased the expression of IL-8, whereas IFNγ had no such effect on HUVECs (Figure 4). Cotreating the cells with rMASP1 and LPS or rMASP-1 and histamine significantly increased the yield of IL-8 secretion, compared to the corresponding individual treatments. Interestingly, cotreatment with rMASP-1 and IFNγ also significantly increased the IL-8 production of HUVECs (Figure 4).

### 2.5. rMASP-1 and LPS Cooperates in Endothelial Permeability Induction

**Figure 5:**
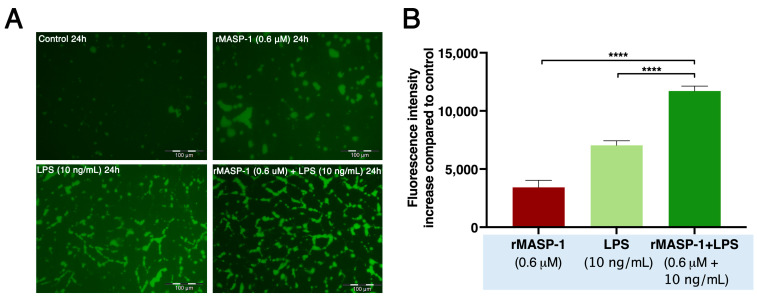
rMASP-1 and LPS cotreatment increased endothelial permeability. Confluent layers of HUVECs were cultured in 96-well plates precoated with biotinylated gelatin. The cells were treated with LPS (10 ng/mL), rMASP-1 (0.6 μM), both, or with medium alone (control) for 24 h. Cell-free areas of the surface of biotinylated gelatin were stained with Streptavidin-Alexa488, and after fixation, fluorescence was detected with a fluorescence plate-reader; representative images were taken using an Olympus IX-81 fluorescence microscope. (**A**) Representative images of three independent experiments. The scale bar applies to all photos. (**B**) Mean values of three independent experiments normalized to the controls. Compared to the control: control vs. rMASP-1: ***; control vs. LPS: ****, control vs. rMASP-1 + LPS: ****; *** *p* < 0.001; **** *p* < 0.0001.

To measure the intensity of paracellular transport through the endothelial layer, we used a modified version of the XperT technique [9,29]. Both 24 h rMASP-1 and LPS treatments themselves increased permeability, but the combination of the two treatments further opened the endothelial layer (Figure 5).

**Figure 6:**
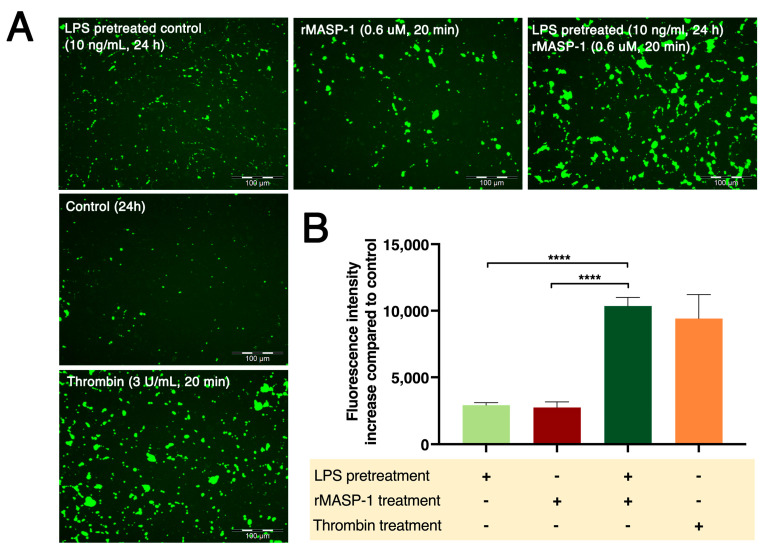
LPS pretreatment followed by rMASP-1 treatment increased endothelial permeability. Confluent layers of HUVECs were cultured in 96-well plates precoated with biotinylated gelatin. The cells were pretreated with LPS (10 ng/mL) or with medium alone (control) for 24 h, and then rMASP-1 treatment (0.6 μM) was added for 20 min. Thrombin (300 nM, 20 min) was used as a positive control. The cell-free areas of the biotinylated gelatin surface were stained with Streptavidin-Alexa488, and after fixation, fluorescence was detected with a fluorescence plate reader; representative images were taken using an Olympus IX-81 fluorescence microscope. (**A**) Representative images of three independent experiments. The scale bar applies to all photos. (**B**) Mean values of three independent experiments normalized to the controls. Compared to the control: control vs. LPS-pretreated control: ****, control vs. rMASP-1: ***, control vs. LPS-pretreated rMASP-1: ****, control vs. thrombin: ****; +: indicates that the pretreatment or treatment was added; -: indicates that pretreatment or treatment was not added; *** *p* < 0.001; **** *p* < 0.0001.

rMASP-1 induced elevated permeability in both the long term (24 h) and the short term (20 min). This dose-dependent increase could be further strengthened by pretreating the cells with LPS (Figure 6).

### 2.6. mRNA Measurements for Receptor Expression

**Figure 7:**
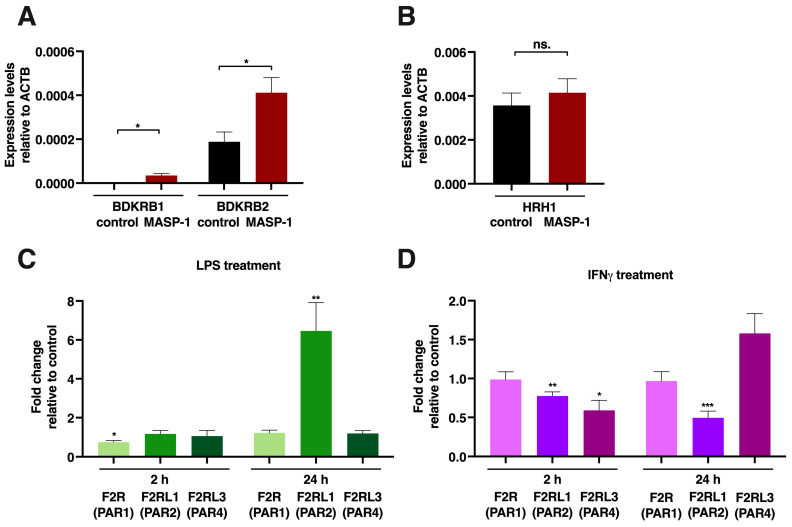
rMASP-1, LPS and IFNγ changed the expression patterns of several endothelial cell receptors. Confluent layers of HUVECs were cultured in 24-well plates, and the cells were treated with rMASP-1 (2 μM), LPS (100 ng/mL), or IFNγ (20 ng/mL) for 2 or 24 h. RNA was purified, and after reverse transcription, quantitative PCR was performed using Qiagen Rotor-Gene Q. β-actin was used as an internal control. (**A**,**B**) Relative expression levels of B1 and B2 bradykinin receptors (BDKRB1, 2) and the histamine H1 receptor (HRH1) after 2 h of rMASP-1 treatment. Data from three independent experiments (**C**,**D**). The effect of LPS or IFNγ treatment on the expressions of PARs 1, 2, and 4. The values represent the ratio of the expression level of treated vs. untreated control. One sample *t*-test was carried out to see if fold changes significantly differed from 1. Data from three independent experiments. * *p* < 0.05; ** *p* < 0.01; *** *p* < 0.001; ns: not significant.

One of the most straightforward modes of cooperation is that pretreatment increases the expression of the receptor for the factor used subsequently. To assess this effect, we measured the expressions of histamine (HRH1), bradykinin (BDKRB1,2), and MASP-1 (PARs 1, 2, and 4) receptors, as the responses given to these were significantly increased by certain pretreatments. As we demonstrated, rMASP-1 pretreatment potentiated the bradykinin-induced Ca^2+^ mobilization in HUVECs (Figure 2B). Along with this, rMASP-1 significantly increased the mRNA levels of both B1 and B2 bradykinin receptors (BDKRB1 and 2) (Figure 7A).

rMASP-1 also increased the sensitivity of endothelial cells to histamine (Figure 2B), and we found a tendency for elevated mRNA levels in the H1 histamine receptor after a 2 h long rMASP-1 treatment, although it was not statistically significant (Figure 7B).

LPS pretreatment potentiated several rMASP-1-induced cellular responses (Ca^2+^ mobilization, E-selectin expression, and permeability); therefore, we measured the mRNA expression of the three protease-activated receptors that could be activated by MASP-1. We found a strong increase in the PAR2 mRNA levels after a 24 h LPS treatment (FC: 6.46), whereas the levels of the other two receptors remained unchanged (Figure 7C). Besides LPS, IFNγ also increased rMASP-1-induced Ca^2+^ mobilization. We found interesting changes in the pattern of PAR receptor expression. The expression of PAR2 was downregulated (2 h FC: 0.77, *p* < 0.01; 24 h FC: 0.49, *p* < 0.001), while the expression of PAR4 slightly decreased after 2 h (FC: 0.59, *p* < 0.05), then slightly increased after 24 h (FC: 1.58; although it did not reach statistical significance, the tendency was clear) (Figure 7D).

## 3. Discussion

Here, we report for the first time that the complement serine protease MASP-1 was able to cooperate with other activators to induce various pro-inflammatory responses in endothelial cells. We showed that the intracellular Ca^2+^ mobilization, the expression of receptors, adhesion molecules and cytokines, and the increased permeability were significantly modified by diverse experimental setups (i.e., different timings and combinations of the activators).

On its own, MASP-1 induced intracellular Ca^2+^ mobilization, the expression of E-selectin and IL-8, and increased the permeability of endothelial cells.

As we are the first to examine the cooperation between MASP-1 and other activators, there is no direct literature data available on this topic. Although information on the use of specific PAR agonists or thrombin is available, it is important to keep in mind that there are differences between MASP-1 and thrombin receptor usage. While thrombin is able to cleave PARs 1, 4, and possibly PAR3 but not PAR2 [30], MASP-1 cleaves PARs 1, 2, and 4 [5].

Cotreating HUVECs with MASP-1 and other activators showed that MASP-1 cooperated with LPS in the induction of IL-8 and E-selectin expression. In line with our findings, Ostrowska and Reiser showed, in airway epithelial cells, that the simultaneous LPS and PAR1 agonist, PAR2 agonist, or thrombin treatment increased the expression of IL-8 more strongly than separate treatments did [31]. This phenomenon may be due to the use of common signaling pathways. In our previous article, we showed that MASP-1-triggered IL-8 production was regulated predominantly by the p38-MAPK pathway [7]. We also showed that LPS induced the p38-MAPK pathway in HUVECs [28]. Besides the activation of p38-MAPK, Rallabandhi et al. showed that both the PAR2 agonist and LPS caused phosphorylation of ERK 1/2 and increased the IL-8 and tissue factor (TF) expression at the mRNA level in SW620 colonic epithelial cells [32]. Pan et al. found that LPS increased Il-8 production via the NFAT pathway in epithelial cells [33], and according to Jia et al., PAR2 receptor activation also promoted the nuclear translocation of NFAT [34].

Pretreatment with MASP-1 followed by adding LPS did not result in any significant changes in the measured responses. However, when we pretreated HUVECs with LPS and then added MASP-1, we observed significant cooperation in Ca^2+^ mobilization, E-selectin expression, and the induction of permeability. Our receptor expression measurements at the mRNA level showed that LPS directly increased PAR2 receptor expression, possibly causing the stronger reactions observed. In line with our findings, Chao et al. found that LPS increased PAR2 expression, as well as Ca^2+^ mobilization; MCP-1 expression and p38 phosphorylation were augmented in cells pretreated with LPS subsequently treated with trypsin [35]. As mentioned above, MASP-1 also activated the p38-MAPK pathway [7], creating the possibility for an additional cooperation at the level of the signaling pathways.

MASP-1 increased IL-8 production, whereas IFNγ treatment alone did not induce the expression of IL-8 in HUVECs. Beck et al. also found that IL-8 production was not affected by IFNγ treatment in HUVECs [36]. Interestingly, the combination of the two treatments significantly increased the production of IL-8. Similar to our results, Suk et al. found that IFNγ enhanced thrombin-induced IL-8 production in the human monocytic cell line U937 [37]. As there are no currently available data on the direct cooperation of the IFN and PAR receptors, we hypothesize that the cooperation occurs at the level of the signaling pathways and/or the transcription factor usage of the IL-8 gene promoter region. The promoter of IL-8 has binding sites for IFNγ-activated (e.g., IRFs, STAT1) and PAR receptor-activated transcription factors (e.g., NFAT, NFκB) [38,39].

Both MASP-1 and histamine alone increased the IL-8 production of the HUVECs. We found that MASP-1 and histamine cotreatment further induced this. Zhou et al. found that histamine induced IL-8 expression via the NFAT pathway in HUVEC [40]. As mentioned above, the activation of PAR2 also promoted the nuclear translocation of NFAT [34], so we hypothesize that the stronger induction of the NFAT pathway can be behind the observed, increased IL-8 production.

MASP-1 also affected the expression of BK receptors. We found an increase in the mRNA levels of both BK receptors (BDKRB1 and 2) after MASP-1 treatment and, in line with this, enhanced Ca^2+^-mobilization to BK.

We did not find a significant increase in the histamine receptor (HRH1) expression at the mRNA level after a 2 h long MASP-1 treatment, but there was a tendency towards it; in line with this, increased Ca^2+^-mobilization to histamine was observed after MASP-1 pretreatment. Since MASP-1 activates the NFκB pathway, and there are several NFκB binding sites in the promoter region of HRH1, it is possible that we can find higher mRNA levels at later time points.

IFNγ pretreatment increased the Ca^2+^ mobilization in response to MASP-1 treatment. In concert with this, we found that IFNγ pretreatment significantly changed the expression pattern of PAR2 and PAR4 at the mRNA level. In line with our findings but using thrombin as a secondary stimulator, Watanabe et al. found that IFNγ pretreatment enhanced the Ca^2+^ mobilization (and prostacyclin production) in HUVECs [41].

**Figure 8:**
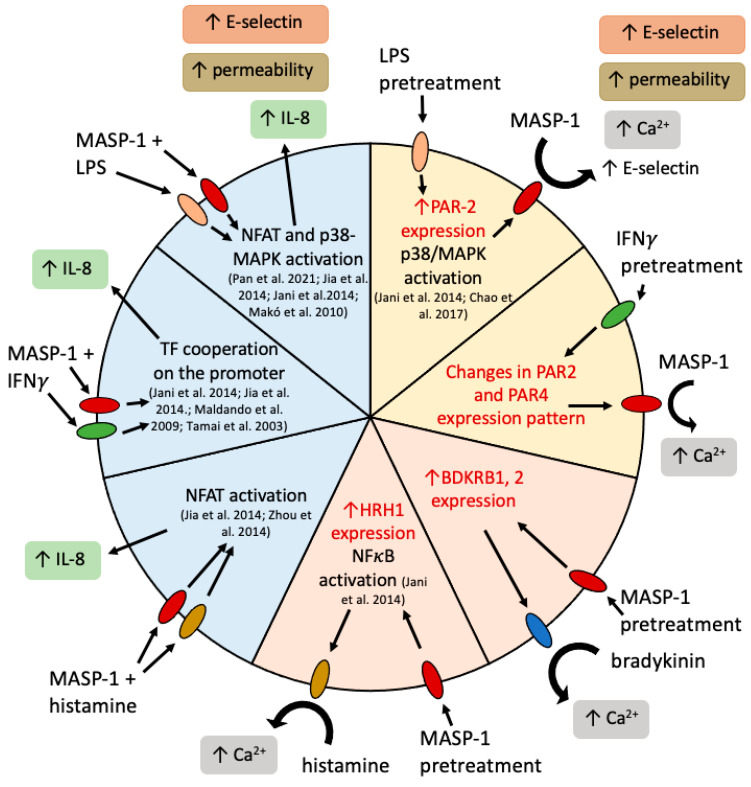
Complement MASP-1 and other proinflammatory factors cooperated in the induction of proinflammatory changes in endothelial cells. A yellow background indicates pretreatment with a coactivator followed by rMASP-1 treatment, a pink background indicates rMASP-1 pretreatment followed by treatment with a coactivator, and a blue background indicates cotreatment. Inside the circle, we indicated possible ways of cooperation. The red text indicates results described in this article; black text indicates data from literature [7,28,34,35,38,39,40]. Blue oval: bradykinin receptors 1 and 2; red oval: PARs 1, 2, and 4; green oval: interferon-γ receptors 1 and 2; golden oval: histamine receptor 1; rose-colored oval: toll-like receptor 4.

It is important to note that our study had limitations. Due to the large number of cooperations studied, we did not have the possibility to thoroughly investigate the possible mechanisms standing behind the observed interaction. We only relied on mRNA measurements and literature data when we tried to find an explanation for them. Our main goal was to identify the diverse types of cooperations and highlight the importance of the combined effect of various activators on the endothelial cells (Figure 8).

In conclusion, here, we present the very first study investigating the cooperation of complement MASP-1 and other inflammatory factors in the induction of proinflammatory changes in endothelial cells. As endothelial cells play a substantial role in the regulation of inflammation by balancing pro- and anti-inflammatory states, minor effects on endothelial cells can cause major inflammatory changes. Despite the use of suboptimal doses of all activators, we found several cases where Ca^2+^ mobilization, the permeability of the endothelial layer, and the expression of cytokines and adhesion molecules were significantly enhanced by the treatments. These cooperations could also lower the threshold of endothelial cells to danger signals by enhancing the receptor expressions. In particular, we found considerable types of cooperation between MASP-1 and LPS, highlighting the risks of bacterial infections under conditions where the complement system was already activated or could be easily triggered.

## 4. Materials and Methods

### 4.1. Reagents

The recombinant catalytic fragment of human MASP-1 (CCP1-CCP2-SP, hereinafter: rMASP-1) was expressed in *Escherichia coli* and purified by the method described by Dobó et al. [42]. rMASP-1 preparations were free of bacterial contaminations and could be inhibited by the C1 inhibitor, as previously described [8,43,44].

All other reagents were purchased from Merck-Sigma-Aldrich (Darmstadt, Germany), unless otherwise stated.

### 4.2. Preparation and Culturing of Human Umbilical Vein Endothelial Cells (HUVECs)

Endothelial cells were harvested from fresh umbilical cords obtained during cesarean sections of healthy neonates by collagenase digestion, as described earlier [45]. HUVECs were grown in gelatin-precoated flasks (Corning, NY, USA) in MCDB-131 medium (ThermoFisher Scientific, Waltham, MA, USA) supplemented with 5% heat-inactivated calf serum (FCS), 2 ng/mL human recombinant epidermal growth factor (R&D Systems, Minneapolis, MN, USA), 1 ng/mL human recombinant basic fibroblast growth factor, 0.3% insulin–transferrin–selenium (ThermoFisher Scientific, Waltham, MA, USA), 1% chemically defined lipid concentrate (ThermoFisher Scientific, Waltham, MA, USA), 1% Glutamax solution (ThermoFisher Scientific, Waltham, MA, USA), 1% penicillin–streptomycin antibiotics solution, 5 μg/mL ascorbic acid, 250 nM hydrocortisone, 10 nM HEPES, and 7.5 U/mL heparin (the completed medium is hereinafter: Comp-MCDB). For some experiments, the medium was replaced with AIM-V medium (ThermoFisher Scientific, Waltham, MA, USA) supplemented with 1% FCS, 2 ng/mL human recombinant epidermal growth factor (R&D Systems), 1 ng/mL human recombinant basic fibroblast growth factor, and 7.5 U/mL heparin (hereafter referred to as Comp-AIM-V).

Each experiment was performed in at least three independent, primary HUVEC cultures from different individuals before passage four. The study was conducted in conformity with the WMA Declaration of Helsinki; its protocol was approved by the Semmelweis University Institutional Review Board (permission number: TUKEB141/2015). All participants provided their written informed consent before inclusion.

### 4.3. Intracellular Ca^2+^ Mobilization Assay

Intracellular Ca^2+^ mobilization was measured using the method described previously [5]. HUVECs were seeded in 96-well plates in 100% confluency and cultured in Comp-MCDB medium for 24 h; then, the medium was changed to Comp-AIM-V for an additional 24 h. The cells were pretreated with the selected agonist in a suboptimal dose (rMASP-1: 0.6 μM, LPS: 10 ng/mL, histamine: 5 μM, IFNγ: 2 ng/mL, or bradykinin: 2 μM) for 24 h or not pretreated. An amount of 2 μM Fluo-4-AM (ThermoFisher Scientific, Waltham, MA, USA) was used to load the cells for 20 min; then, the cells were incubated in HBSS for another 20 min. Measurements were carried out with fluorescence microscopy; sequential images were taken every 5 s. To determine baseline fluorescence, two images were taken before adding the treatment. Twenty cells per image were analyzed using Olympus CellP 2.1 software.

### 4.4. Measurement of E-Selectin and VCAM-1 Expression by Cell-Based ELISA

HUVECs were cultured in 96-well plates at 100% confluency in Comp-MCDB medium for 24 h. They were then pretreated with the selected agonist at suboptimal doses or not pretreated. After 24 h of pretreatment, the cells were treated with agonists for E-selectin measurement for 6 h or for VCAM-1 measurement for 24 h. The cells were fixed and stained with mouse anti-human E-selectin (ThermoFisher Scientific, Waltham, MA, USA) or mouse anti-human VCAM-1 (BD Biosciences, Franklin Lakes, USA) antibodies at room temperature for 1 h. Then, HRP-conjugated goat anti-mouse antibody (Southern Biotech, Birmingham, AL, USA) and 3,3′5,5′-tetra methyl benzidine (TMB) were used to measure the expression of adhesion molecules.

### 4.5. Measurement of IL-8 Cytokine Production by Sandwich ELISA

HUVECs were seeded in 96-well plates at 100% confluency and cultured in Comp-MCDB medium for 24 h. They were then pretreated with the selected agonist at suboptimal doses or not pretreated. After 24 h of pretreatment, the cells were treated with the agonists for 24 h, and the supernatants were collected. Supernatants were diluted 1:20, and IL-8 was measured using the sandwich ELISA kit (R&D Systems, Minneapolis, MN USA) according to the manufacturer’s instructions.

### 4.6. Permeability Measurement

Permeability tests were carried out using a modified version of the XPerT technique, as described earlier [9]. Briefly, HUVECs were seeded in 96-well plates precoated with biotinylated gelatin at 100% confluency and cultured in Comp-AIMV medium. After various pretreatments and treatments, Streptavidin-Alexa488 (ThermoFisher Scientific, Waltham, MA, USA) was added to each well for 2 min. The cells were fixed with 1% paraformaldehyde–PBS, and a fluorescence plate reader (Tecan Infinite M1000 Pro) was used to quantify the fluorescence. Representative images of each well were also taken using an Olympus IX-81 fluorescence microscope.

### 4.7. RNA Purification and Quantitative Real-Time PCR

HUVECs were cultured in 24-well plates to 100% confluency in Comp-MCDB medium, then treated with IFNγ (20 ng/mL), bradykinin (20 μM), or LPS (100 ng/mL) for 2 or 24 h. RNA isolation was carried out using the illustra™ RNASpin RNA Isolation Kit (GE Healthcare, Chicago, CA, USA) according to the manufacturer’s protocol. RNA–cDNA transcription was performed with the Tetro cDNA Synthesis Kit (Bioline, Essex, UK). SensiFAST SYBR Master Mix—No ROX Kit (Bioline) was used for the quantification of cDNA using a Rotor-Gene Q (Qiagen, Hilden, Germany) real-time PCR cycler. Primers (Table 2) were designed using the NCBI Primer-BLAST primer design tool and synthetized using IDT (Coralville, IA, USA). The purity and size of PCR products were checked by sequencing (sequencing was performed by Biomi Ltd., Gödöllő, Hungary) after the first use of each primer pair and by high-resolution melting curve analysis for each measurement.

Quantification was performed using the Rotor-Gene Q Pure Detection 2.1.0 software (Qiagen, Hilden, Germany), and values of interest were normalized with that of β-actin.

### 4.8. Statistical Analysis

Experiments were performed in duplicate (for Ca^2+^ mobilization assay and mRNA measurement) or triplicate (for adhesion molecule, cytokine, and permeability measurements) and repeated at least three times using HUVECs from different individuals. Statistical analysis was performed using Student’s *t*-test, one-sample *t*-test, or one-way ANOVA with GraphPad Prism 8.4.0 software (GraphPad). A *p* ≤ 0.05 was considered statistically significant. Data are presented as means ± SEM unless otherwise stated.

## Figures and Tables

**Table 1:** Cooperation between rMASP-1 and other coactivators. Various combinations of pretreatments and cotreatments were applied on the HUVECs, and several indicators of the endothelial cell activation were measured. An interaction was considered significant if the measured outcome of the combined treatment was statistically greater or smaller than the effect of each activator alone in the same experimental setup. All significant interactions (*p* < 0.05) that we found were positive (indicated by green coloring), and their directions are indicated by arrows.

			Interaction				
	Pretreatment	Treatment	Ca^2+^-Mobilization	Permeability	E-Selectin	VCAM-1	IL-8
**Cotreatment**		rMASP-1 + LPS		**↑**	**↑**		**↑**
		rMASP-1 + Histamine					**↑**
		rMASP-1 + IFNγ					**↑**
		rMASP-1 + Bradykinin					
**Pretreatment with rMASP-1**	rMASP-1	LPS					
	rMASP-1	Histamine	**↑**				
	rMASP-1	IFNγ					
	rMASP-1	Bradykinin	**↑**				
**Pretreatment with coactivator**	LPS	rMASP-1	**↑**	**↑**	**↑**		
	Histamine	rMASP-1					
	IFNγ	rMASP-1	**↑**				
	Bradykinin	rMASP-1					

**Table 2:** The primers used in the qPCR reactions.

	Gene Name		Sequence
β-actin (ACTB)		forward	5′-ATCAAGATCATTGCTCCTCCTGA-3′
		reverse	5′-AAGGGTGTAACGCAACTAAGTCA-3′
B1 bradykinin receptor (BDKRB1)		forward	5′-CACAGAGTGCTGCCAACATTTAT-3′
		reverse	5′-ACTGGTTCCAGATATTCTCTGCC-3′
B2 bradykinin receptor (BDKRB2)		forward	5′-TCTGAGTCCAAATGTTCTCTCCC-3′
		reverse	5′-AGGACAAAGATGTTCTCTAGGGTG-3′
Histamine H1 receptor (HRH1)		forward	5′-GTCTTCATCCTGTGCATTGATCG-3′
		reverse	5′-AAGTCTGTCTCACACTTGTCCTC-3′
Proteinase-activated receptor 1 (F2R)		forward	5′-CTGTGTACACCGGAGTGTTTGT-3′
		reverse	5′-AGTAAAATGCTGCAGTGACGAA-3′
Proteinase-activated receptor 2 (F2RL1)		forward	5′-AAGAGGGCCATCAAACTCATT-3′
		reverse	5′-GTTCTTTGCATGATCCCTGAA-3′
Proteinase-activated receptor 4 (F2RL3)		forward	5′-ACCATGCTGCTGATGAACCT-3′
		reverse	5′-AGCACTGAGCCATACATGTGAC-3′

## Data Availability

Not applicable.
